# Dynamics of calling activity to toll-free numbers in China

**DOI:** 10.1371/journal.pone.0230592

**Published:** 2020-03-26

**Authors:** Jun Gui, Zeyu Zheng, Dianzheng Fu, Zihao Yang, Yuan Gao, Zhi Liu

**Affiliations:** 1 Shenyang Institute of Automation, Chinese Academy of Sciences, Shenyang, China; 2 Institutes for Robotics and Intelligent Manufacturing, Chinese Academy of Sciences, Shenyang, China; 3 Key Laboratory of Network Control System, Chinese Academy of Sciences, Shenyang, China; 4 University of Chinese Academy of Sciences, Beijing, China; 5 School of Information Science and Engineering, Shenyang University of Technology, Shenyang, China; Beihang University, CHINA

## Abstract

The quantitative understanding of human behavior is a key issue in modern science. Recently, inhomogeneous human activities have been described by bursts (consecutive activities separated by long periods of inactivity) and characterized by fat-tailed inter-event time (interval between two activities) distributions. However, the dynamics between number of activities and activity duration are still unclear. In this study, we analyzed 133 million toll-free call records from China to study the dynamics between call frequency and call duration. We confirmed that both call frequency and call duration exhibit circadian cycles and weekly cycles. By analyzing intraday patterns of these two metrics, we found the opposite volatility and clustered distributions. Results of clustering analysis showed that calling activity to toll-free numbers can be clustered into four clusters. In the “Work” cluster, the distribution of call duration was significantly different from that in the other clusters. The corresponding time of “Work” cluster was much shorter than estimates based on common sense. Intraday patterns and clustering results showed that both call frequency and call duration are primarily related to circadian cycles, the nature of human beings, and that work is a secondary factor that affects these variables. Moreover, we found a strong positive correlation between call frequency and call duration, as well as polarization of joint probability. The polarization indicates two extremes in inhomogeneous calling activity to toll-free numbers, i.e., either people are very busy or very idle. The empirical probability of the extreme was approximately four times that of random probability. Our findings may have great usage for studying the dynamics of inhomogeneous human behavior.

## Introduction

In the era of “Big Data”, Call Data Records (CDRs) have been increasingly used to study social physics [1] and socioeconomics [2]. These CDRs-based studies have already been conducted from different perspectives, including social network, human mobility, and human dynamics [1, 2]. Studies on social network have discovered static structures [3–5], dynamic structures [6, 7], temporal motifs [8, 9], and evolution of individual and group behavior [10–13], which has helped us understand and model human society. From another perspective, studies on human mobility have been focusing on patterns [14–18], models [19, 20], prediction [21], and correlation between human mobility and social relationship [22–24], making it possible to understand and predict human mobility. In addition to these two perspectives, in the last decade, human dynamics have been receiving attention.

Studies on dynamics of behaviors such as calling [7, 25–29] and sending short messages [30, 31] have revealed that the inter-event time (interval between two activities) exhibits a non-poisson distribution, which is consistent with the findings of studies on other human behaviors such as email communication [32–34] and online activities [35–38]. There are three popular mechanisms used to explain the non-poisson distribution of inter-event time; decision-based queuing process [32], adaptive interests [39], and poisson processes modulated by circadian and weekly cycles [33, 34]. A recent study proved that the non-poisson distribution of inter-event time in mobile phone communication activity are affected by circadian and weekly cycles as well as by correlations rooted in human task execution [29]. Evidently, the time factor must be taken into consideration while studying human dynamics. Besides the inter-event time, the duration of an activity is also related to many social phenomena, such as long queues at a supermarket, blocked communication network, and crowed public traffic. This led us to the exploration of dynamics of calling activity from another perspective.

Call frequency and call duration are two key metrics used to describe human calling activity. The aggregated duration between two users was used to describe the strength of a tie to build a social network [7, 40]. In previous studies, call frequency has been used to describe the emotional closeness of two individuals [13], the in-degree and the out-degree of an Ego Communication Network [41], the calling activity in a given hour of a given day to study dynamics of human resting or sleeping patterns [42], and individuals’ daily patterns [43]. Call duration has been examined to study relationships between aggregated time spent on the phone, personal network size, tie strength, and the way in which users distributed their limited time across their network [40], and to describe different gender specific patterns in calling activity [43]. Although call frequency and call duration have been commonly used to study human behavior, the dynamics between these two metrics have not been clearly revealed. Further, the dynamics of calling activity, as described by these two metrics have not been discovered either.

In this paper, we use call frequency and call duration to describe calling activity instead of inter-event time, to study the dynamics of calling activity to toll-free numbers in China. Moreover, considering the findings of previous studies [29, 33, 34], we also examine the time-related characteristics of calling activity to reveal the dynamics more clearly. Commonly, daily patterns and intraday patterns are examined to discover time-related characteristics of human behavior, such as calling activity between individuals [43], web browsing activity [35], user activity in virtual worlds [36], Wikipedia editorial activity [37], activity on Twitter and Instagram [38], and spatio-temporal properties of cities in Spain [44]. Motivated by these studies, we apply the k-means method and statistical analysis to examine daily patterns and intraday patterns.

## Results

The present dataset on toll-free call records provided by a telecommunication provider in China contained the time and duration of 133,181,465 toll-free calls from Jan. 1. 2014 to Dec. 31. 2014, allowing us to investigate calling activity to toll-free numbers throughout the year. We used call frequency and average call duration in a given day and in a given hour of each day to describe daily calling activity and intraday calling activity, respectively. This paper presents the results pertaining to the analysis of daily and intraday patterns.

### Daily pattern

#### Weekly cycles

**Fig 1:**
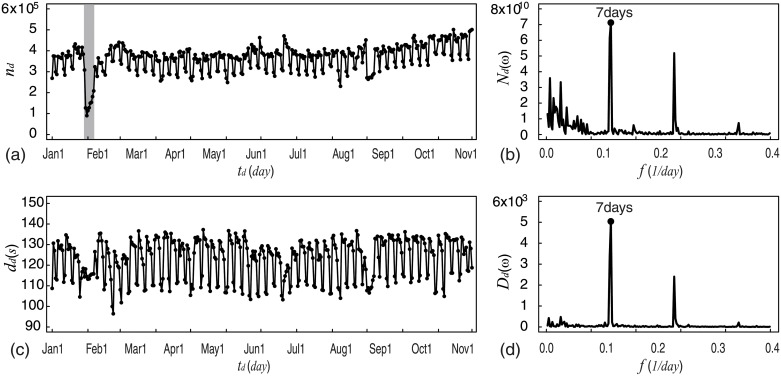
Weekly cycles. We found weekly cycles in both daily patterns of call frequency and average call duration. (a) Daily pattern of call frequency (*n*_*d*_) appears as a periodic fluctuation, with a peculiarity on Spring Festival (gray area). (b) Spectrum density (*N*_*d*_) shows that the period of *n*_*d*_ is one week. (c) Daily pattern of average call duration (*d*_*d*_) appears as a periodic fluctuation. (d) Spectrum density (*D*_*d*_) shows that the period of *d*_*d*_ is also one week.

We began by examining the calling activity over the entire observation period and investigated the calling activity by analyzing daily patterns. Fig 1 shows the time series and spectrum density of call frequency and average call duration. As evident in Fig 1(a), *n*_*d*_ fluctuates from 230,503 to 501,441 (white area). In Fig 1(b), spectrum density (*N*_*d*_) shows that the period of *n*_*d*_ is one week. Interestingly, *n*_*d*_ on the Spring Festival (gray area in Fig 1(a)), the most important traditional festival in China, was significantly lower than that on other days. In Fig 1(c), *d*_*d*_ fluctuates from 100 to 140 and in Fig 1(d), spectrum density (*D*_*d*_) shows that the period of *d*_*d*_ is also one week. We noticed that both *n*_*d*_ and *d*_*d*_ fluctuate along the same period (one week). Similarly, several other studies on human behavior have reported a similar weekly cycle [16, 21, 33, 34, 36, 37, 43].

#### Clustering characteristic

**Fig 2:**
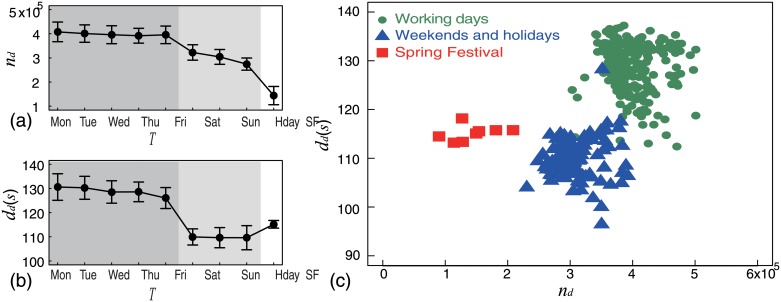
(Color online) Clustering characteristic. We found a time-related clustered distribution of daily calling activity. (a) Error (Standard Deviation) bar curve shows three levels of call frequency (*n*_*d*_) related to working days (from Monday to Friday), weekends and holidays (Hday), and Spring Festival (SF). (b) We used the same method to draw the error bar curve for the average call duration (*d*_*d*_). The curve also shows three levels related to date. (c) Points represent daily calling activity described by call frequency (x-axis) and average call duration (y-axis). The scatter plot shows a clear clustering feature. People make more and relatively longer calls on working days (green circles), fewer and relatively shorter calls on weekends and holidays (blue triangles), and the fewest calls on Spring Festival (red squares, the most important festival in China).

Since we discovered that both call frequency and average call duration are related to time and that they exhibit weekly cycles, to further investigate calling activity, we classified dates into nine categories; the seven days of the week (from Monday to Sunday), a holiday (Hday), and Spring Festival (SF). Subsequently, we computed the mean and standard deviation for each category. As shown in Fig 2(a), we used the error bar curve to explore the relationship between call frequency and date. We observed three levels. People made the most number of calls on working days (from Monday to Friday, dark gray area), fewer calls on weekends and holidays (Hday, light gray area) than working days, and the fewest calls on Spring Festival (SF, white area). We also noticed an obvious decreasing tendency of call frequency from Saturday to Spring Festival. This seems to indicate that the longer the rest time is, the fewer calls people prefer to make. Then we used the same method to explore the relationship between average call duration and date. Again, three levels were observed, as evident from Fig 2(b). Specifically, people preferred to make longer calls on working days and shorter ones on rest days. However, one peculiarity was that people made longer calls on Spring Festival than on weekends and holidays, although they made the fewest calls on Spring Festival. Unlike call frequency, we noticed an obvious decreasing tendency in call duration from Monday to Friday. This seems to indicate that the closer to the weekend, the shorter are the calls.

Similar three date-related levels were evident in Fig 2(a) and 2(b), leading us to explore the clustering characteristics using a two-dimension scatter plot. Fig 2(c) shows a clustered distribution, which indicates that people make more and relatively longer calls on working days (green circles), fewer and relatively shorter calls on weekends and Holidays (blue triangles), and the fewest calls on Spring Festival (red squares). The clustering characteristic indicates the polarized distribution of daily calling activity. To describe this clustered distribution further, we examined the statistical properties of call frequency and call duration. These findings have been presented in the next section.

#### Statistical properties

**Fig 3:**
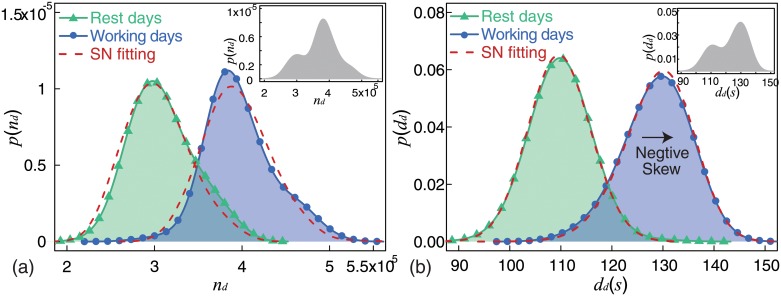
(Color online) Statistical properties. We found that only the distribution of average call duration (*d*_*d*_) on working days (blue circle point curve in (b)) was a negative skew distribution, while the other three distributions were positive skewed. All four distributions can be fitted by skew normal distributions (See Table 1). (a) Empirical distributions of call frequency (n_*d*_) on working days (blue circle point curve) and rest days (green triangle point curve). Red dashed lines show the best fit to the empirical distributions. The inset shows the empirical distribution of call frequency without partitioning working and rest days. (b) Empirical distribution of average call duration (*d*_*d*_) on working days (blue circle point curve) and rest days (green triangle point curve). Red dashed lines show the best fit to empirical distributions. The inset shows the empirical distribution of the average call duration without partitioning working and rest days. Estimated distribution parameters and results of the KS test have been presented in Table 1.

This section presents the findings on the analysis of the empirical distributions of call frequency and average call duration. Fig 3(a) shows the probability density curves of call frequency on working days (blue circle point curve) and on rest days (green triangle point curve). Evidently, these are normal-like distributions. Azzalini pointed out that normal-like distributions can be described by Skew-Normal distributions, SN(*ϵ*,*ω*,*α*) [45]. To find the best fit, we estimated distribution parameters by using the Maximum Likelihood Estimation (MLE) method and applied the Kolmogorov-Smirnov (KS) test. These findings have been presented in Table 1. By using the estimated parameters, we drew the best fitting curves (red dashed lines) in Fig 3(a). As evident from the KS statistics in Table 1, the empirical distributions can be described by Skew-Normal distributions. The most significant difference between these two distributions is *ϵ*, indicating different calling activities described by two distributions with similar shapes but different locations. The inset in Fig 3(a) is the empirical distribution of call frequency without partitioning working and rest days. It is difficult to explain why the distribution is bimodal, and we could not quantify the different distributions of call frequency on working days and rest days.

**Table 1:** Estimated distribution parameters and KS test results for daily calling activity.

Empirical Data	*ϵ*	*ω*	*α*	KS Statistic	*P*-Value
*n*_*d*_ rest days	270126.9	52132.4	2.83	0.092	0.74
*n*_*d*_ working days	360280.3	52263.6	2.12	0.068	0.60
*d*_*d*_ rest days	106.8	5.1	1.15	0.065	0.98
*d*_*d*_ working days	135.0	8.1	-5.66	0.081	0.40

We used the same method to find the best fitted distributions for average call duration. Fig 3(b) shows the probability density curves for average call duration on working days (blue circle point curve) and rest days (green triangle point curve). Estimated distribution parameters and KS test results have been shown in Table 1. These two distributions can also be described by Skew-Normal distributions. However, unlike call frequency, we notice that these two distributions are quite different. One significant difference is *ϵ*. The other significant difference is *α*. These two distributions indicate different calling activities described by two distributions with different locations and shapes. The inset in Fig 3(b) is the empirical distribution of the average call duration without partitioning working and rest days. It is difficult to explain why the distribution is bimodal, and we could not quantify the different distributions of average call duration on working and rest days.

In this section, we describe the clustering characteristic and statistical properties of calling activity by analyzing daily patterns. We found that human activity is also influenced by circadian cycles [29, 43], and therefore, we analyzed intraday patterns of calling activity. These findings have been presented in the next section.

### Intraday pattern

#### Distribution of hourly calling activity

In last section, we examined daily patterns. In this section, we further investigate calling activity by analyzing intraday patterns. We defined *t*_*h*_ as a series of hours in 2014. For each hour, we calculated the call frequency and average call duration, and then we defined *n*_*h*_ as a series of call frequency and *d*_*h*_ as a series of average call duration. Here, we present intraday patterns described by scatter plots. The intraday patterns display not only the relationship between calling activity and circadian cycles represented by 24 hours, but also the distribution of calling activity in a certain hour. Considering the analysis results of daily patterns, we examine the intraday pattern of working and rest days.

**Fig 4:**
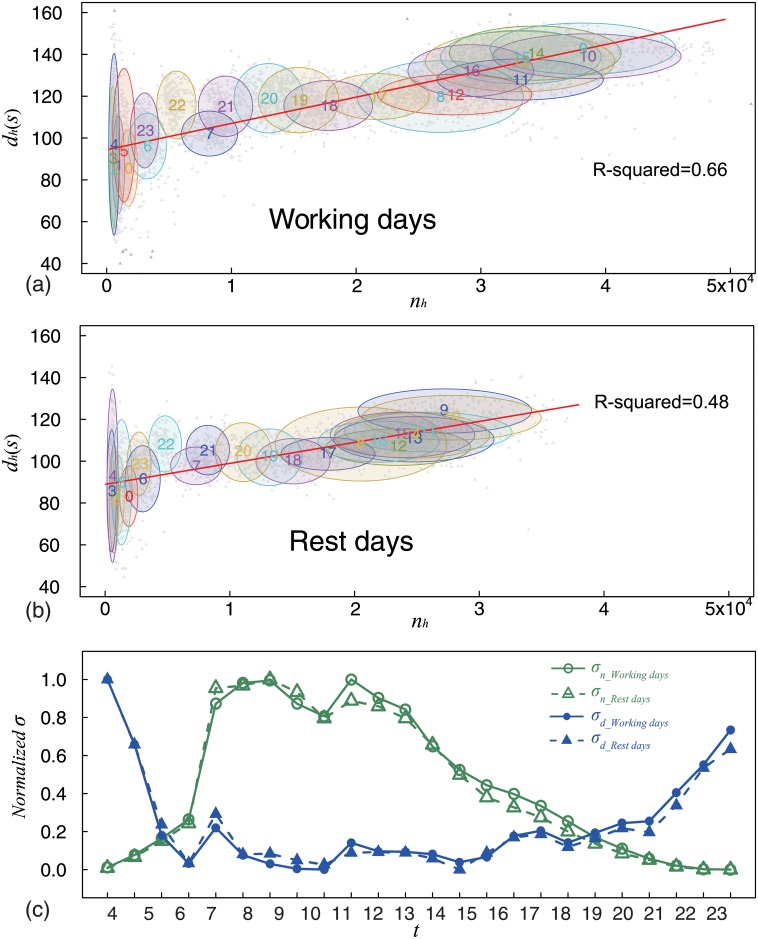
(Color online) Intraday patterns. We found a strong positive correlation between average call duration and call frequency, and almost the opposite intraday pattern for standard deviations. (a) Here, we use a scatter plot to show intraday pattern of working days. Each point represents calling activity described by call frequency (x-axis) and average call duration (y-axis) in a given hour of a given working day. The ellipses indexed by numbers show the distribution areas for 24 hours. The locations and shapes of ellipses show three distribution features (details see main text) related to circadian cycles. The red solid linear regression line with R-squared = 0.66 indicates a strong positive correlation. (b) We used the same method to present the intraday pattern for rest days. Again, these findings showed the same distribution features as those found in (a) and a strong positive correlation between call frequency and average call duration. (c) Intraday patterns of standard deviation of call frequency (green solid line with circles for working days, green dashed line with triangles for rest days) and average call duration (blue solid line with circles for working days, blue dashed line with triangles for rest days). Overlapping features of lines show that standard deviations are only related to circadian cycles. Lines with different colors show that the standard deviations for intraday patterns of call frequency and call duration are almost opposite.

Fig 4(a) shows the intraday pattern for working days. Each point represents the calling activity described by call frequency (x-axis) and average call duration (y-axis) in a given hour of a given working day. The ellipses indexed by numbers show distribution areas for 24 hours. The center coordinates of each ellipse are determined by the mean values of all points in a given hour. The height is four times the standard deviation of the average call duration and the width is four times the standard deviation of the call frequency. Each ellipse covers 88.7%-93.5% points in a given hour. The locations and shapes of ellipses show three features. The overlapped ellipses in the left area indicate that people make the fewest calls at midnight, and the dispersion degree of call frequency is smaller than that of the average call duration. The ellipses in middle area indicate that people make a moderate number of calls in the morning and evening, and that the dispersion degree of the call frequency and average call duration is similar. The overlapped ellipses in the right top area indicate that people make the most calls and prefer to make longer calls during daytime, and that the dispersion degree of the call frequency is larger than that of the average call duration. Subsequently, we used the Least Square Method to fit the linear regression of call frequency and average call duration on working days. The fitted Intercept was 0.00097 and fitted coefficient was 89.5. The R-squared was 0.66 (correlation coefficient was 0.81), indicating a strong positive correlation between call frequency and average call duration on working days.

Fig 4(b) shows the intraday pattern for rest days. It was drawn using the same method as that described for working days. Each ellipse covers 88.0%-95.4% points in a given hour. The intraday pattern for rest days shows the same features as those found in Fig 4(a), indicating that these features are intrinsic and are related to circadian cycles. We used the same method to fit the linear regression of call frequency and average call duration on rest days. The fitted Intercept was 0.0012 and fitted coefficient was 94.2. The R-squared was 0.48 (correlation coefficient was 0.69), indicating a strong positive correlation between call frequency and average call duration on rest days. Furthermore, by comparing the two intraday patterns presented in Fig 4(a) and 4(b), we observed that the locations of the same time period (not at midnight) were different, thus suggesting that calling activity is also related to working or rest days. The different locations of ellipses show that people prefer to make more and longer calls during daytime on working days. The overlapped ellipses indicate similar calling activity at midnight, irrespective of whether they were working or rest days.

The width of each ellipse is determined by the standard deviation of call frequency in a given hour. The height of each ellipse is determined by the standard deviation of average call duration in a given hour. The standard deviation is usually used to describe the variability of a group of samples [46]. Here, we explored the variability of days in a given hour by analyzing the intraday patterns of standard deviations. Opposite intraday patterns for call frequency and average call duration are evident from in Fig 4(c). We found the following time-related features. There were two critical time points, 6:00 and 22:00. At these two time points, all standard deviations are equal, indicating the same variability. Before and after these two time points, standard deviations are greatly different, indicating different variability. Several studies have reported similar intraday patterns for web browsing behavior [35], activity in the virtual world [36], and calling activity [29, 43]. Intraday patterns for the average call duration are almost the opposite to these reported patterns. These intraday patterns show that the dispersion degree varies with physiological status determined by the circadian cycles. Furthermore, the intraday patterns of working and rest days are the same, indicating that the dispersion degree is not related to working or rest days. We find that variability of the call frequency is proportional to the physiological activity level, and the variability of the average call duration in the same observation period is inversely proportional to the physiological activity level.

We also observed that some ellipses are similar and overlapped in Fig 4(a) and 4(b). These similar and overlapped ellipses led us to investigate the clustering characteristic, as described in the next section.

#### Four states

**Fig 5:**
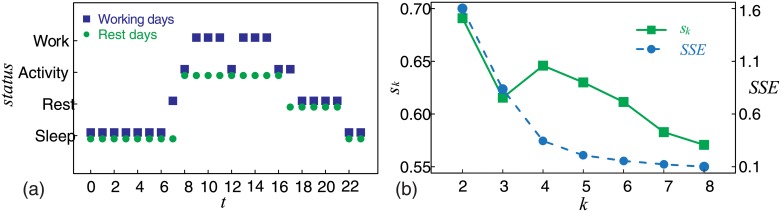
(Color online) Four clusters. We found that calling activity can be clustered into four clusters and we identified the corresponding time of each cluster. We also found that the duration of “Work” was only six hours, which is substantially shorter than the eight hours that professionals are expected to work per working day in China. (a) The scatter plot shows the clustering results; four states (y-axis) and corresponding time (x-axis). Blue squares represent working days. Green circles represent rest days. (b) We drew a within-cluster Sum of Squared Errors (SSE) curve (blue dashed line with circles) and *S*_*k*_ (Silhouette Coefficient) curve (green solid line with squares) to choose the number of clusters (*k*). These two curves show that the most appropriate number of clusters was four.

We used the K-means method to investigate the clustering characteristic implied by intraday patterns. The clustering results presented in Fig 5(a) revealed four clusters that are consistent with four states of our lives; “Sleep,” “Rest,” “Activity,” and “Work.” The clusters “Sleep,” “Rest,” and “Activity,” show that calling activity is related to circadian cycles. However, “Work” and “Activity” clusters show that calling activity is also related to work or rest. Additionally peculiarities were observed. At 7:00 clock on working days, the state of calling activity was “Rest,” while it was “Sleep” at 7:00 clock on rest days. At 17:00 clock on working days, the state of calling activity was “Activity,” while it was “Rest” at 17:00 clock on rest days. The most significant peculiarity was the difference between “Work” and “Activity.” China implements an eight-hour work system in which people are required to work for eight hours per day. However, the duration of the “Work” state was only six hours. Therefore, we further investigated the statistical properties of the four states. The related results have been presented in the next section.

#### Statistical properties

**Fig 6:**
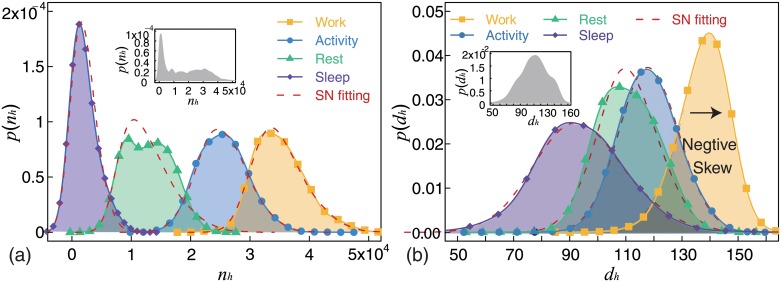
(Color online) Statistical properties. We found that only the distribution of the average call duration (*d*_*h*_) in “Work” (yellow distribution in (b)) was a negative skew distribution, while the other seven distributions were positive skew distributions. (a) Empirical distributions of call frequency (*n*_*h*_) in four states, “Sleep” (purple distribution), “Rest” (green distribution), “Activity” (blue distribution), and “Work” (yellow distribution). Red dashed lines are the best fits. The inset shows the distribution of call frequency without partitioning based on the four states. (b) Empirical distribution of average call duration (*d*_*h*_) in four states; Sleep (purple distribution), Rest (green distribution), Activity (blue distribution), and Work (yellow distribution). Dashed lines are the best fits. The inset shows the distribution of average call duration without partitioning based on the four states. Estimated distribution parameters and results of the KS test have been presented in Table 2.

In this section, we present the findings of the analysis of the empirical distributions of call frequency and average call duration. Fig 6(a) shows the probability density curves for call frequency for the four states of “Sleep” (purple distribution), “Rest” (green distribution), “Activity” (blue distribution), and “Work” (yellow distribution). These four distributions were normal-like. Additionally, we calculated distribution parameters by using the MLE method and KS test, the results of which have been presented in Table 2. Red dashed lines in Fig 6(a) are fitting curves. KS statistics showed that the two distributions of call frequency in “Activity” and “Work” were Skew-Normal distributions, while the other two were not. The empirical distribution of call frequency in “Sleep” (purple distribution) was leptokurtic and it differed substantially from the other three distributions. This indicates the difference between “Sleep” and the other three states. The empirical distribution of call frequency in “Rest” (green distribution) was platykurtic, which indicates the diversity of call frequency in this state. The distribution in “Activity” (blue distribution) and “Work” (yellow distribution) had similar shapes. The most significant difference between these two distributions was (*ϵ*). The inset in Fig 6(a) is the empirical distribution of call frequency without partitioning according to the four states. It is difficult to model such a distribution. As compared to the inset, the four fitted distributions with different shapes and locations are clearer and more accurate in quantifying the distributions of call frequency.

**Table 2:** Estimated distribution parameters and KS test results for four states.

Empirical Data	*ϵ*	*ω*	*α*	KS Statistic	*P*-Value
*n*_*h*_ in “Sleep”	0	2587.5	124.8	0.101	≈0
*n*_*h*_ in “Rest”	7591.5	6334.7	5.7	0.090	≈0
*n*_*h*_ in “Activity”	21892.3	5367.7	1.46	0.026	0.52
*n*_*h*_ in “Work”	30390.9	6334.7	2.54	0.023	0.81
*d*_*h*_ in “Sleep”	82.6	19.4	1.06	0.014	0.88
*d*_*h*_ in “Rest”	103.9	11.9	0.99	0.056	0.01
*d*_*h*_ in “Activity”	113.6	10.5	0.65	0.027	0.47
*d*_*h*_ in “Work”	146.3	11.6	-2.68	0.015	0.99

Fig 6(b) shows the probability density curves for the average call duration in four states; “Sleep” (purple distribution), “Rest” (green distribution), “Activity” (blue distribution), and “Work” (yellow distribution). These four distributions were also normal-like. Estimated distribution parameters and KS test results have been presented in Table 2. Red dashed lines in Fig 6(b) are fitting curves. Three empirical distributions were Skew-Normal distributions. The *ω* of “Sleep” was approximately 2 times those of the other three states. This indicates the diversity in call duration and dispersion distribution in the “Sleep” (purple distribution) state. The distribution in “Rest” (green distribution) was similar to that in “Activity”(blue distribution). Further, the distribution in “Work” (yellow distribution) was substantially different from those in the other three states. One significant difference was *ϵ*. The other significant difference was the skew. The distribution in “Work” (yellow distribution) was a negative skew, while those in the other three were positive skews. The inset in Fig 6(b) is the empirical distribution for the average call duration without partitioning based on the four states. The distribution had only one obvious peak and hardly described the four states of calling activity. Compared to the inset, the four fitted distributions with different shapes and locations were clearer and more accurate for quantifying the distributions of average call duration.

### Polarized distribution

**Fig 7:**
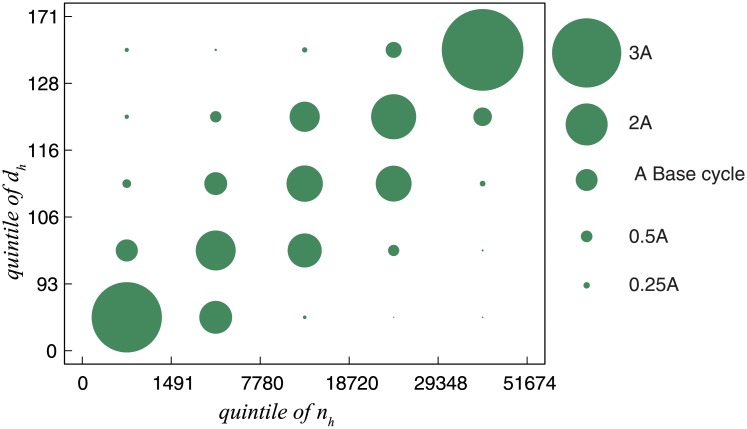
(Color online) Polarized joint probability. The x-axis gives quintiles of call frequency (n_*h*_). The y-axis gives quintiles of call duration (d_*h*_). The radius of green circles are proportional to the joint probability *P*(*n*_*h*_*i*_, *d*_*h*_*j*_)(*i,j* = 1…5). The radius of the base cycle is calculated and denoted by A, assuming uniform distribution. Two largest circles on the top right and bottom left indicate polarized distribution. The circles on the diagonal are the largest on each row or column, indicating a strong positive correlation.

The different distributions of the four states presented in Fig 6 imply a centralized distribution of call frequency and average call duration in a specific state. To extending this, we examined the joint probability to investigate the concurrence of call frequency and average call duration. Fig 7 shows the *P*(*n*_*h*_*i*_, *d*_*h*_*j*_), the distribution of hours broken up into quintiles of call frequency and average call duration (*i,j* = 1…5). The x-axis shows quintiles of call frequency and the y-axis shows the quintiles of average call duration. We calculated the radius of the base cycle, denoted by A (*P* = 0.04), assuming that the 8736 data points are equally distributed into 5 × 5 = 25 sets of approximately 350 points each. Essentially, Fig 7 is a heat map with circle size being used in lieu of color intensity. The polarized distribution is quite evident. The joint probability for the top right quintiles *P*(*n*_*h*_5_, *d*_*h*_5_) = 0.16 was four times the radius of Base cycle. The joint probability for the bottom left quintiles *P*(*n*_*h*_5_, *d*_*h*_5_) = 0.14 is also approximately four times the radius of the base cycle. In contrast, the joint probabilities for the top left quintiles or the bottom right quintiles are approximately one-quarter the radius of the base cycle. Polarization indicates two extremes in calling activity to toll-free numbers that people are either very busy or very idol. Further, we found that the joint probabilities on the diagonal are the largest on each row or column, indicating a strong positive correlation between call frequency and average call duration.

## Discussion

Call records have been proven to be an important source of information regarding human dynamics. In this paper, we have presented the findings of a study on calling activity to toll-free numbers in China during one year. By analyzing daily patterns, we confirmed weekly cycles. This finding is in agreement with those of previous studies on other human behavior such as email communication or activity in the virtual word. By examining the scatter plot for call frequency and call duration, we found that people prefer to make more and longer calls on working days. In addition, by applying statistical tests, we found that call frequency and call duration can be described by Skew-Normal distributions.

After analyzing daily patterns, we focused on intraday patterns. By comparing distribution shapes and positions, we found that hourly calling activity is primarily related to intraday time and secondarily related to work or rest. Moreover, we applied the K-means method to identify similarities in calling activity. We found that calling activity to toll-free numbers can be described by four states of our lives: “Sleep,” “Rest,” “Activity,” and “Work.” We were surprised by the finding that the time length of the “Work” state was much shorter than our common sense estimate. We also found that both call frequency and call duration can be described by Skew-Normal distributions.

We found a strong positive correlation between call frequency and average call duration, which was evident for both working and rest days. However, the different R-squared values on working and rest days indicates that the correlation is stronger on working days. This correlation also seems to be primarily related to intraday time and secondarily related to work or rest. Further, the same intraday patterns of standard deviation on working and rest days shows that the variability of days is not related to work or rest, and that it is only related to circadian cycles. Weekly and circadian cycles have been reported by many studies on human behaviors such as web browsing behavior [35], activity in the virtual world [36], and calling activity [29, 43]. The intraday patterns in call frequency observed in the present study were similar with those reported in these studies; however, the intraday patterns of average call duration were quite different. This difference may be explained by common and individual needs. For instance, people may make a large amount of calls for similar common needs during daytime, causing low variability in call duration. In contrast, they make a small amount of calls for different individual needs at night, causing a large variability in call duration. Furthermore, the duration of the “Work” state was only six hours. Our finding shows that the first and last hour of work was clustered into the “Activity” state. In China, many cities adopt the system of staggered working hours to avoid traffic jams. Therefore, people start and finish work at different times, which may have caused the present results.

Further, different distributions of the four states showed that people prefer to make longer calls when they make more calls. We examined this preference by analyzing joint probability. The joint probability distribution presented in Fig 7 shows the polarization of call frequency and average call durations. The joint probability of the largest call frequency and largest average call duration was approximately four times that of the even distribution. This polarized distribution implies that, when people make the most calls in a given hour, they spend the longest time on these calls. This preference is called burstiness, which has been observed in several studies on inter-event time of human behavior [7, 25–39]. Three popular mechanisms are used to explain the burstiness of human behavior. Our results support the mechanism of poisson processes modulated by circadian and weekly cycles [33, 34], instead of the decision-based queuing process [32] or adaptive interests [39]. The inter-event time only represents the interval between two consecutive activities. Here, we used two metrics to describe calling activity to toll-free numbers. Our work shows that these two metrics can be used to characterize inhomogeneous human behavior. Our findings may better explain some social phenomena, such as the tendency of people to feel much busier when they are focusing on a series of similar jobs, long queues at the supermarket, blocked communication network, and crowed public traffic.

## Materials and methods

### Data

In this work, we analyzed a dataset provided by a telecommunication provider in China. For billing purposes, the telecommunication provider collects all calling records for toll-free number separately. Our dataset only contains call records for toll-free numbers. None of the call records in our dataset were collected from communication towers. Instead, they were provided by the data center. We were informed that the dataset was used to generate bills for all registered toll-free numbers. The time span of the dataset was Jan. 01, 2014 to Dec. 31, 2014. For unknown reasons, records for Dec. 01 2014 were missing. The dataset comprised 133,181,465 records. Interesting attributes were “Calling number,” “Called number,” “Starting time,” “Duration,” and “Area.” For privacy protection, the last several bits of the “Calling number” were erased. Calls were made from 344 cities covering all major cities in China. In total, 240,591 toll-free numbers were called, with one specific number being called 12,566,638 times. Further, 61,829 toll-free numbers were called only once.

### Data preprocessing

We aimed to analyze daily and intraday patterns of call frequency (represented by *n*) and average call duration (represented by *d*). To analyze daily patterns, we defined *t*_*d*_ as a series of dates in 2014. For each day, we calculated call frequency and average call duration, and then we defined *n*_*d*_ as a series of call frequency and *d*_*d*_ as a series of average call duration. To analyze intraday patterns, we defined *t*_*h*_ as a series of dates and hours in 2014 and calculated *n*_*h*_ and *d*_*h*_, respectively.

### Estimation of distribution parameters

To find the best fit, we estimated distribution parameters and applied statistical tests. Azzalini [45] introduced density functions of Skew-Normal distribution to describe normal-like distributions. If a random variable *X* has the following density function:

$$\begin{array}{c} f(x)=2\phi (x)\Phi (\alpha x)\;(-\infty <x<\infty ) \end{array}$$ (1)

where *ϕ* and Φ are the standard normal density and distribution function, respectively, we say that *X* is a Skew-Normal random variable with parameter *α*. For applied work, *location* and *scale* parameters must be introduced. The variable

$$\begin{array}{c} Y=\epsilon +\omega x \end{array}$$ (2)

will be called a Skew-Normal (SN) variable with *location* parameter *ϵ*, *scale* parameter *ω*, and *shape* parameter *α*. Its density function is

$$\begin{array}{c} \frac{2}{\omega }\phi (\frac{x-\epsilon }{\omega })\Phi (\alpha \frac{x-\epsilon }{\omega })\equiv \frac{1}{\omega }\phi (\frac{x-\epsilon }{\omega };\alpha ) \end{array}$$ (3)

and we say *Y* ∼ *SN*(*ϵ*, *ω*^2^, *α*).

We used the Maximum Likelihood Estimation (MLE) method to estimate the *ϵ*, *ω*, and *α* of empirical distributions. Estimated parameters have been presented in Tables 1 and 2. The Kolmogorov–Smirnov test (KS test) is a nonparametric test of equality of continuous, one-dimensional probability distributions that can be used to compare two samples. We applied the KS test to examine empirical data and to fit data with estimated distribution parameters. Results of the KS test have also been presented in Tables 1 and 2.

### Clustering based on distribution similarity

We used an ellipse to describe the distribution of calling activity in a given hour of working or rest days. There were 48 ellipses corresponding to 24 hours of working days and 24 hours of rest days. Each ellipse was described by four parameters; the mean values of call frequency and call duration, and the standard deviation of call frequency and call duration. We observed the similarities in these distributions, and then used the K-means method to explore the clustering characteristics of calling activity. We calculated a series of SSE (within-cluster Sum of Squared Errors) and a series of *S*_*k*_ (Silhouette Coefficient) for different cluster numbers (from 2 to 8). Silhouette Coefficient provides an evaluation of clustering validity, and might be used to select an “appropriate” number of clusters [47]. For each cluster number *k*,*S*_*k*_ is defined in the following form:

$$\begin{array}{c} s\left(i\right)=\frac{b(i)-a(i)}{max\{a(i),b(i)\}} \end{array}$$ (4)

$$\begin{array}{c} S_{k}=\frac{\sum_{i=1}^{n}s\left(i\right)}{n} \end{array}$$ (5)

where *a*(*i*) is the average distance between object *i* to all other objects in the same cluster, *b*(*i*) is the minimum average distance between object *i* to all objects in other clusters, and *n* is number of objects. Detailed results have been presented in the subsection Four States.
